# Hamartome sébacé de la face: à propos d’un cas

**DOI:** 10.11604/pamj.2018.30.132.14911

**Published:** 2018-06-14

**Authors:** Jawad El-Azhari, Mohammed Boui

**Affiliations:** 1 Service de Dermatologie-Vénérologie, Hôpital d’Instruction Mohammed V, Rabat, Maroc

**Keywords:** Hamartome sébacé, Naevus sébacé, la face, adulte, Sebaceous hamartoma, sebaceous naevus, face, adult

## Abstract

Sebaceous naevus (sebaceous hamartoma) is a congenital lesion mainly localized to the scalp, whose clinical and histologic diagnosis is generally easy. In the vast majority of cases, it is diagnosed at birth, but it can sometimes go unnoticed and give the impression to occur later. This lesion may be complicated by benign tumors or more rare malignancies. We report the case of a 45-year old patient, who had been followed-up for Biermer's anemia (treated with monthly HYDROXOCOBALAMINE injection) and ophiasic alopecia for 15 years, presenting with a 10-year history of bilateral and symmetric pigmented plaques gradually increasing in size. Clinical examination showed temporal and subauricular plaques, with brownish verrucous appearance and smooth surface (A, B). Histological examination objectified acanthosis, papillomatosis and voluminous sebaceous glands located abnormally high within the dermis, suggesting sebaceous hamartoma. CO_2_ laser treatment was proposed to the patient.

## Image en médecine

Le nævus sébacé (ou hamartome sébacé) est une lésion congénitale localisée principalement au cuir chevelu, dont le diagnostic clinique et histologique est généralement aisé. Il est diagnostiqué dès la naissance dans la grande majorité des cas, mais peut parfois passer inaperçu et donner l'impression d'apparaître plus tardivement. Cette lésion peut se compliquer de tumeurs bénignes ou de façon plus rare malignes. Nous rapportons le cas d'un patient de 45 ans, suivi depuis 15 ans pour anémie de Biermer (traitée par injection mensuelle d'HYDROXOCOBALAMINE) et une pelade ophiasique, et qui consulte pour des plaques pigmentées apparues il y'a plus de 10 ans; bilatérales et symétriques, augmentant progressivement de taille. A l'examen clinique, il s'agit de plaques temporales et sous-auriculaires, d'aspect verruqueux brunâtre à surface lisse (A, B). L'étude histologique objectivait une acanthose, une papillomatose, et des glandes sébacées volumineuses et situées anormalement hauts dans le derme, en faveur d'un hamartome sébacé. Un traitement par laser CO_2_ a été proposé au patient.

**Figure 1:**
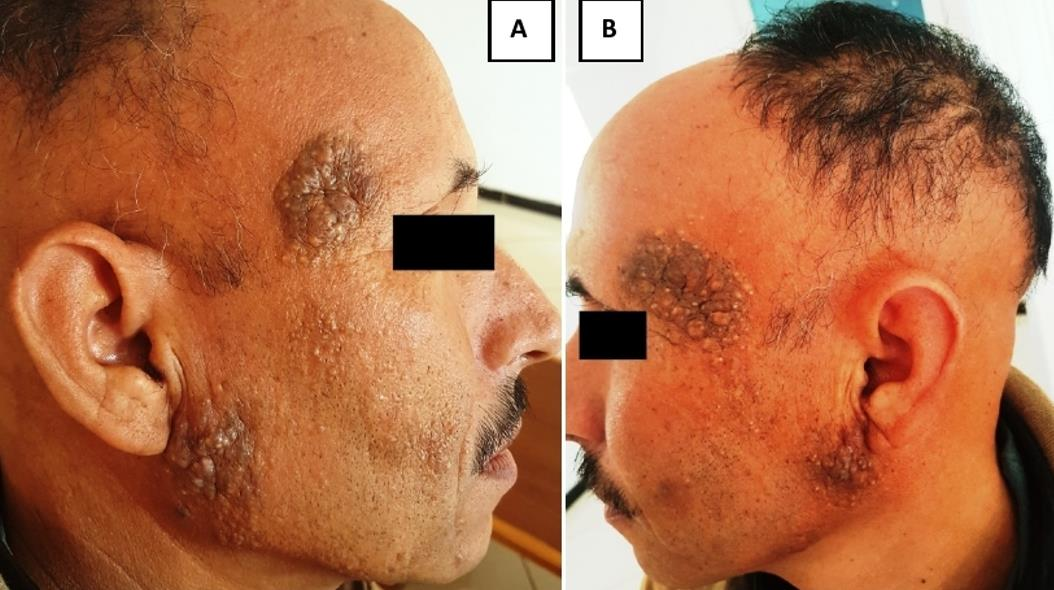
Plaques temporales et sous-auriculaires, bilatérales et symétriques

